# Association between peripheral markers in women with malaria in pregnancy and small newborns: A cross-sectional study

**DOI:** 10.1371/journal.pgph.0005526

**Published:** 2025-12-03

**Authors:** Maria Inês dos Santos, Alexsander Rodrigues Carvalho Júnior, Gabriela Mendes de Oliveira, Laura Cordeiro Gomes, Wasim Aluísio Prates Syed, Erika Paula Machado Separovic, Sabrina Epiphanio, Gerhard Wunderlich, Cláudio Romero Farias Marinho, Jamille Gregório Dombrowski

**Affiliations:** 1 Department of Parasitology, Institute of Biomedical Sciences, University of São Paulo, São Paulo, Brazil; 2 Department of Immunology, Institute of Biomedical Sciences, University of São Paulo, São Paulo, Brazil; 3 Department of Clinical and Toxicological Analysis, Faculty of Pharmaceutical Sciences, University of São Paulo, São Paulo, Brazil; Indian Institute of Public Health Shillong, INDIA

## Abstract

Alterations in angiogenic proteins play a role in newborn growth potential, and they have been widely studied, but not further explored in malaria research. We aimed to identify maternal peripheral proteins that are associated with newborn growth parameters in the context of malaria in pregnancy. We analysed data and biological samples collected from 386 pregnant women with and without malaria selected from previous cohort study in the Amazon region, Brazil, between 2013 and 2015. ELISAs were used to quantify Ang-1, Ang-2, Tie2, VEGF, sFlt1, VEGFR2, PlGF, sENG, and leptin in peripheral plasma at time of delivery. We generated receiver operating characteristic curves and multivariable logistic regression models to assess the associations of maternal peripheral protein levels with the anthropometric profiles of newborns. Tie2, sFlt1, sENG and leptin were associated to growth parameters in newborns of mothers who had malaria during pregnancy. sFlt1 levels were associated with newborn weight and length, and sENG was associated with newborn length in *Plasmodium vivax*-infected women. Tie2, sFlt1 and leptin levels were associated with newborn length in *Plasmodium falciparum*-infected women. Identifying maternal peripheral proteins associated with newborn outcomes might help in understanding biological dynamics of malaria in pregnancy in areas of low transmission, improving future health interventions, and encourage further studies with angiogenic proteins.

## Author summary

During pregnancy, women undergo significant physiological changes, including alterations in the levels of proteins essential for maintaining a healthy pregnancy. In the event of infections, such as those caused by *Plasmodium* spp., these natural changes can be altered, potentially leading to complications for both mother and baby. Although placental markers are commonly studied due to their direct role in foetal development and the placental environment, it is important to recognize that protein alterations can also occur systemically. This raises the question: which maternal peripheral proteins could be associated with changes in newborn size in the context of malaria in pregnancy? In this study, we analysed nine angiogenic markers in maternal peripheral blood at delivery and their association with newborn anthropometric parameters. Our findings revealed that maternal plasma levels of Tie2, sFlt1, sENG, and leptin were associated with reduced birth size parameters. Given that physiological changes vary throughout pregnancy, our analysis specifically focuses on the third trimester, a critical period for foetal growth and development.

## Introduction

Malaria in pregnancy (MiP) represents an important worldwide public health concern that is linked to a series of adverse maternal and child outcomes [1,2]. In 2020, an estimated 121 million pregnant women worldwide and 6.7 million women in the Americas were at high risk of acquiring malaria and developing complications [3]. In addition to increasing the risk of miscarriage, stillbirth, death in the first year of life, and low birth weight (LBW) related to prematurity and foetal growth restriction (FGR), MiP can cause severe anaemia and maternal death [4–9]. Placental insufficiency associated with hypoxia is one of the main causes of FGR, which is related to maternal infection in pregnancy [10,11]. FGR puts foetuses at higher risk of neonatal complications such as hypoxia, death [5,10], respiratory problems in childhood, infection [12], chronic diseases in adulthood [13,14], and may also affect brain development and function, causing cognitive and neurological deficits [12,13].

FGR refers to foetuses who do not reach the expected growth potential for their gestational age and differ from the small for gestational age (SGA), which is defined as a newborn weight below the 10^th^ percentile [11]. However, not all SGA babies are growth restricted, with some being small but otherwise healthy newborns [11,13,15]. Nevertheless, growth parameters at birth can give an insight of the development in intrauterine environment and outcome in later life [16].

Angiogenic factors related to placental insufficiency might be key components for identifying pregnant women at risk for FGR complications because of the involvement of these factors in placental vascular development [10]. Among the numerous markers involved in angiogenic functions, angiopoietins 1 and 2 (Ang-1 and Ang-2), their Tie2 receptor, vascular endothelial growth factor (VEGF) and its receptors, placental growth factor (PlGF), endoglin, and leptin stand out. Ang-1 and Ang-2 are growth factor antagonists, both signalling through the Tie-2 receptor; Ang-1 acts in vessel maturation and stabilization, while Ang-2 destabilizes the vasculature to promote vascular remodelling [17]. VEGF and its receptors are critical factors in angiogenesis, controlling the proliferation, migration, and survival of endothelial cells, which facilitates the development of new blood vessels - a process essential for embryonic growth [18]. PlGF plays a similar role, promoting the proliferation, migration, and survival of endothelial cells, acting in a balanced manner through binding to VEGF receptors [19]. Endoglin is a transmembrane protein expressed by epithelial cells and syncytiotrophoblasts, acting together with transforming growth factor-β (TGFβ); however, its soluble peptide (sENG) has an antiangiogenic role, inhibiting the formation of new blood vessels [20]. Leptin is considered an important molecule with multiple functions; in the reproductive system, it acts in the regulation of gonadotropin production, blastocyst formation and implantation, normal placentation, and foeto-placental communication, regulating placental functions [21].

Alterations in these factors can negatively affect the maternal-foetal passage of nutrients, oxygen, and the disposal of waste products [11]. Several proteins from the placenta can be released into the maternal circulation including PlGF and sFlt1 [19]. Some angiogenic proteins have been widely studied in women with preeclampsia and are associated with FGR [14,22]. Maternal circulating angiogenic proteins such as sENG, PlGF, sFlt1 and the sFlt1/PlGF ratio are altered in cases of FGR throughout the three gestational trimesters [11].

In the context of malaria, previous studies have shown that an increase in peripheral Tie2/Ang-1 and Ang-2/Ang-1 ratios and a decrease in Ang-1 levels are associated with MiP [17,23]. Also, decreased Ang-1 levels were associated with increased placental barrier thickness [23]. Furthermore, these alterations also extend to *P. vivax*-infected women, with reduced placental plasma levels for Ang-2, sFLT1, and leptin, and higher levels of Tie2 [8]. Despite the important role of these markers for angiogenesis during pregnancy, few studies have explored the association between maternal peripheral proteins with newborn anthropometric parameters during malaria in pregnancy [24]. Maternal angiogenic and proangiogenic proteins may play a key role in understanding how MiP can affect the growth status of the foetus [10,22]. Based on that, we chose to explore nine peripheral maternal proteins that has been shown to be associated with MiP, PM, or adverse outcomes. In this study, we aim to identify potential maternal peripheral angiogenic proteins associated with newborn growth parameters such as weight, length, and head circumference in the context of MiP.

## Methods

### Ethics statement

Ethical clearance was provided by the committees for research of the University of São Paulo (Plataforma Brasil, CAAE: 03930812.8.0000.5467), according to resolution no. 466/12 of the Brazilian National Health Committee. All subjects or their legal guardians (if minors) gave written informed consent. The study was conducted in accordance with the Good Clinical Practice Guidelines and the Declaration of Helsinki.

### Study site and population

Data used here were originally collected during a cohort study conducted in an endemic area for malaria in Brazil from January 2013 to April 2015 [25]. A total of 600 pregnant women were recruited at the Hospital da Mulher e da Criança do Juruá (HMCJ) in Cruzeiro do Sul, Juruá Valley, Acre. Pregnant women were followed up throughout pregnancy as described previously [8,25]. Malaria during pregnancy was first diagnosed from thin and thick blood smears by microscopic analysis, carried out by the Juruá Valley endemic surveillance team. Subsequently, all samples were further screened by molecular diagnosis using the real-time PCR technique [8,25]. Clinical data and maternal peripheral blood samples, as well as newborn anthropometric measurements, were collected at the time of delivery. All individuals sign an informed consent before participating in the original study and data was anonymized by the study’s principal investigators.

For this study, the data were accessed on 14/10/2020 and the exclusion criteria included alcohol use and/or smoking during pregnancy, history of hypertension or preeclampsia, other infections, twin pregnancies, babies with congenital anomalies, gestational age was not defined by ultrasonography in the first trimester, and less than 27 weeks pregnant (S1 Fig). This selection of samples only in the third trimester (>27 weeks) was performed to minimize bias from expected normal changes in markers during pregnancy [11,17]. The study followed the Strengthening the Reporting of Observational Studies in Epidemiology (STROBE) [26] reporting guidelines (S1 STROBE Checklist).

### Anthropometric measurements

The newborns’ anthropometric information, including weight, head circumference, and length, was obtained by trained nurses within 24 hours after delivery. A digital paediatric scale with a precision of 5 g was used to measure weight in grams (g), and a nonstretching flexible measuring tape was used to assess head circumference and length in centimetres (cm). To classify small newborns, we considered birth weight, length, and head circumference below the 10^th^ percentile, according to the standards of the Intergrowth 21^st^ project, which consider foetal sex and gestational age [27].

### Peripheral protein measurements

The quantification of maternal plasma proteins at delivery was performed using an enzyme-linked immunosorbent assay (ELISA) sandwich in duplicates - samples and assay controls (DuoSet ELISA, R&D Systems - https://www.rndsystems.com) according to the supplier’s instructions with some adaptations (S1 Table). Briefly, 96 well plates (Corning®, United States) were coated with anti-human capture antibody for each protein and incubated overnight at room temperature for 12–18 h. The plates were washed three times, and five times for VEGF and sENG with Phosphate-Buffered Saline (PBS) supplemented with 0.05% Tween, blocked with 1% Bovine serum albumin (BSA) in PBS. Plasma samples were diluted after standardization for each marker, Ang-1 (1:20), Ang-2 (1:10), Tie2 (1:20), VEGF (1:10), sFlt1 (1:5), VEGFR2 (1:10), PlGF (1:5), soluble endoglin (sENG) (1:50), and leptin (1:50). Then, the plates were washed, incubated with biotinylated detection antibody developed using 1:1 Hydrogen peroxide and Tetramethylbenzidine, and the reaction was stopped with 2 N sulfuric acid. Optical density was determined with an ELISA reader (CLARIOstar Plus, BMG Labtech) at a wavelength of 450 nm and 570 nm, as established by the manufacturer.

### Statistical analysis

Clinical and epidemiological data were analysed using the Stata 14.2 software StataCorp LLC, United States). Continuous variables are reported as means and standard deviations, medians, and interquartile ranges (IQRs). Categorical variables are reported as frequencies and percentages. Chi-square tests were used for proportions, and to compare means between groups, we used the Mann-Whitney and Kruskal-Wallis with post-hoc tests. We identified that data were missing in some variables and, for some proteins measurements, levels of detection were out of range. To handle those, we used a complete case analysis for each variable to reduce bias or wrong assumptions by excluding missing data. A two-tailed *p* value less than 5% (*p* < 0.05) was considered statistical significant with a 95% confidence interval (95% CI). Associations between explanatory variables and covariates were also explored by correlation and multivariate logistic regression analyses, controlling for confounding factors, such as maternal age bellow 18 years old, education less than 4 years, rural residence, primigravidae, and gestational age. Protein performance and its association with adverse outcomes were evaluated using receiver operating characteristic (ROC) curves. The variation in the sensitivity and specificity of each protein was assessed, with a minimum area under the curve (AUC) of 70% for quality tests for the final models. The Youden’s index (J = max [sensitivity + specificity - 1]) was used to determine protein cut-offs.

## Results

### Study population and baseline characteristics

Among the 600 pregnant women enrolled in the original investigation, 386 were selected for this study after applying the inclusion and exclusion criteria (S1 Fig). Of these, 169 women had no *Plasmodium* spp. infections, 150 were infected with *P. vivax*, and 67 were infected with *P. falciparum*.

Overall, the median maternal age was 22 years (IQR: 18–27), with infected pregnant women being younger than those non-infected (Table 1). Most of the women had history of malaria, 197 (90.8%) *P. vivax-* and *P. falciparum*-infected women reported that they had a history of malaria in their lifetime, whereas only 92 (54.4%) non-infected women had this background (*p* < 0.001). Moreover, 40 (31.7%) infected women reported a history of MiP, whereas 7 (7.9%) non-infected women reported a history of MiP (*p* < 0.001). There was no difference in the gestational age, newborn weight, or newborn length at birth between the groups (Table 1).

**Table 1:** Characteristics of the study population stratified by infection status.

Characteristics	NI (169)	Mal (217)	*p* ^a^	*Pv* (150)	*Pf* (67)	*p* ^b^
Age, IQR	22 [18-28]	20 [17-26]	0.002	21 [17–26] ^c^	21 [17-27]	0.005
Primigravidae, n (%)	80 (47.3)	91 (41.9)	0.39	66 (44.0)	25 (37.3)	0.57
Previous malaria, %	92 (54.4)	197 (90.8)	<0.001	131 (87.3)	66 (98.5)	<0.001
Previous MiP, %	7 (7.9)	40 (31.7)	<0.001	24 (28.6)	16 (38.1)	<0.001
Gestational age at birth, mean (SD)	39.4 (1.7)	39.1 (2.0)	0.16	39.2 (1.9)	39.0 (2.3)	0.36
Sex - male, n (%)	77 (45.6)	110 (51.4)	0.26	79 (53.4)	31 (47.0)	0.36
Birth weight (Kg), IQR	3.26 [2.98-3.53]	3.13 [2.86-3.49]	0.05	3.13 [2.85-3.51]	3.13 [2.91-3.39]	0.14
Length [cm], mean (SD)	49.0 (2.2)	48.8 (2.2)	0.13	48.8 (2.2)	49.0 (2.2)	0.31
HC [cm], mean (SD)	34.2 (1.6)	33.7 (1.7)	0.01	33.8 (1.6)	33.4 (2.0)^c^	0.01

For proportions, the chi-square test was used, and for differences between groups, the ^a^Mann-Whitney test and the ^b^Kruskal-Wallis test with Dunn’s post hoc test were used. ^c^*P value* <0.05 in comparison to the NI group. Abbreviations: NI, non-infected; Mal, malaria; *Pv*, *Plasmodium vivax*; *Pf*, *Plasmodium falciparum*; IQR, interquartile range; MiP, malaria in pregnancy; HC, head circumference. Three newborns in the Mal group did not have sex data at birth.

### Proteins associated with infection and the anthropometric profiles of newborns

**Fig 1:**
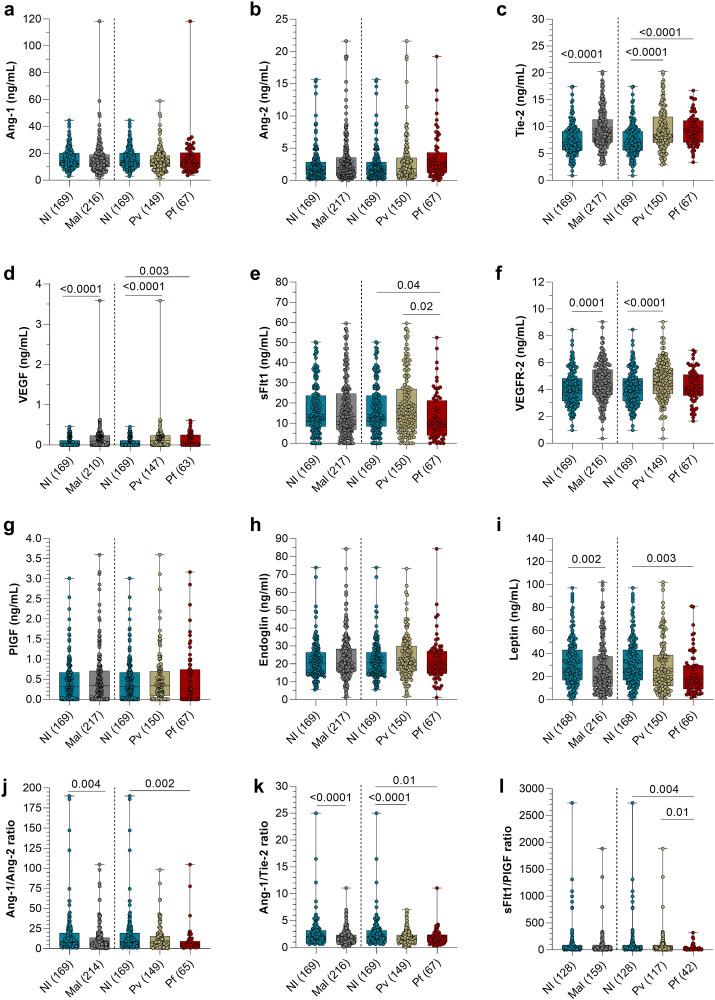
Levels of maternal peripheral plasma proteins from the 27^th^ week of gestation according to the infection status at delivery. Data are presented with a boxplot plot where the upper and lower whiskers indicate the minimum and maximum; the central bar, the median; and dots are observations. Differences and the *p* value between groups NI and Mal were determined by Mann-Whitney and between NI, *Pv*, and *Pf* groups were determined by Kruskal-Wallis test with Dunn’s post-test. Abbreviations: NI, non-infected; Mal, malaria, malaria; *Pv*, *P. vivax*; *Pf*, *P. falciparum*; Ang, angiopoietin; Tie, tyrosine kinase; VEGF, vascular endothelial growth factor; PlGF, placental growth factor. The dashed line indicates the division of the statistical analysis groups, in which on the left are the NI and Mal groups; and on the right are the *Pv* and *Pf* subgroups that were compared with the NI group.

Analysis of the protein levels in maternal peripheral plasma (Fig 1 and S2 Table) revealed increased Tie2 and VEGFR2 levels and decreased Ang-1/Tie2 ratios in *P. vivax*-infected women compared with those non-infected (Fig 1). *P. falciparum*-infected women had the greatest changes, with an increase in Tie2 levels and decrease in sFlt1, leptin, Ang-1/Ang-2, Ang-1/Tie2, and sFlt1/PlGF ratio values compared with the non-infected group (Fig 1). sFlt1 levels were lower in the *P. falciparum* group than in the *P. vivax* group (Fig 1E).

**Fig 2:**
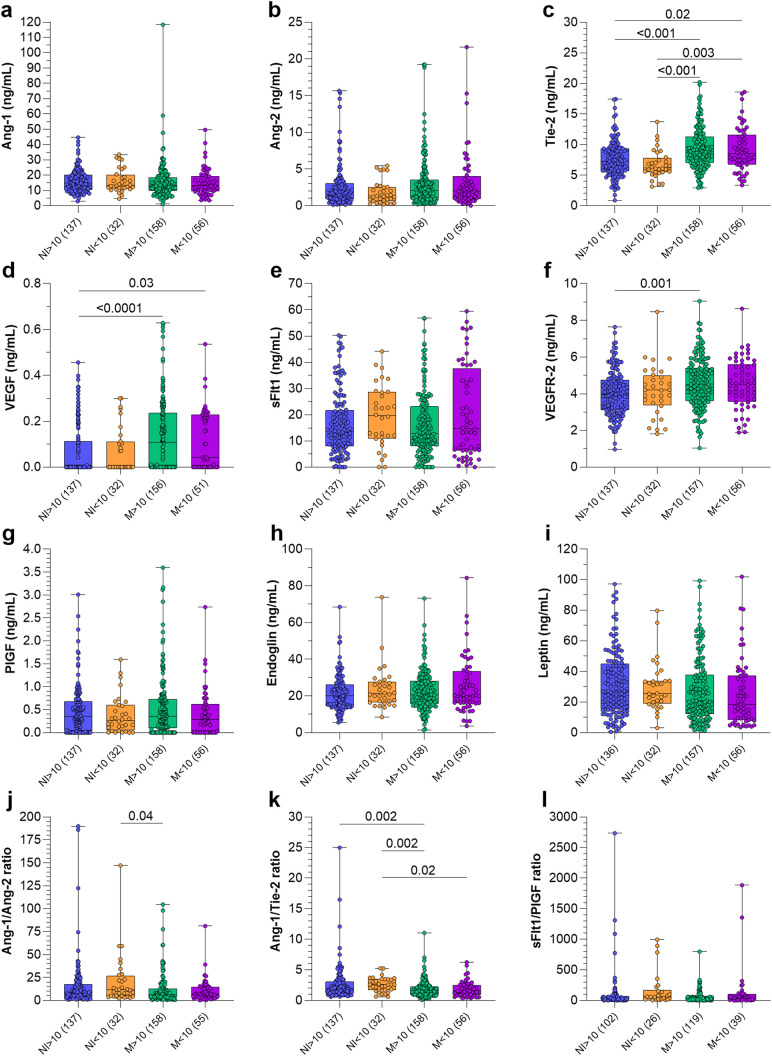
Levels of maternal peripheral plasma proteins according to the size of the baby from the 27^th^ week of gestation at delivery. Data are presented with a boxplot plot where the upper and lower whiskers indicate the minimum and maximum; the central bar, the median; and dots are observations. Differences and the *p* value between groups were determined by Kruskal-Wallis test with Dunn’s post-test. Abbreviations: AGA, adequate for gestational age; FGR, foetal growth restriction; NI, non-infected; M, malaria; Ang, angiopoietin; Tie-2, tyrosine kinase; VEGF, vascular endothelial growth factor; sFlt1, soluble receptor 1 of VEGF; VEGFR2, soluble receptor 2 of VEGF; PlGF, Placental Growth Factor. NI > 10, non-infected women with newborn sized above the 10^th^ percentile; NI < 10, non-infected women with newborn sized below the 10^th^ percentile; M > 10, infected women with newborn sized above the 10^th^ percentile; M < 10, infected women with newborn sized below the 10^th^ percentile.

Additionally, peripheral levels of angiogenic factors were evaluated according to newborn size (Fig 2 and S2 Table). Infected women with newborns independent of the newborn size had an increase in Tie2 compared to both non-infected groups (Fig 2C) and higher levels of VEGF compared to the non-infected women with newborn with adequate size (Fig 2D). Infected women with babies above the 10^th^ percentile had higher levels of VEGFR2 in comparison to the non-infected women with the same newborn profile (Fig 2F), and lower Ang-1/Ang-2 ratio compared with the non-infected group that had newborns under the 10^th^ percentile (Fig 2J). Finally, Ang-1/Tie2 ratio were lower in the infected group with newborns below the 10^th^ percentile compared to the non-infected with the same newborn characteristics, and lower levels in the infected group with normal size newborns compared to both non-infected groups (Fig 2K).

**Fig 3:**
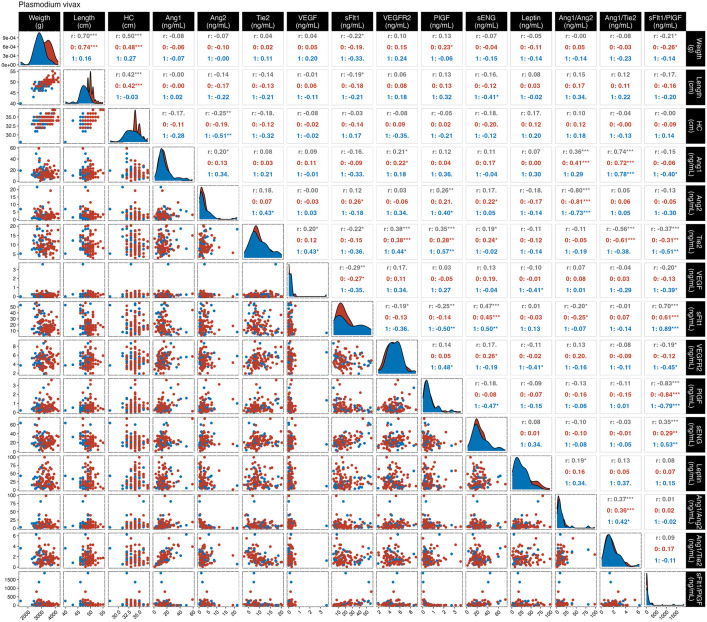
Correlation between maternal peripheral proteins with newborn growth status in the *P. vivax*-infected group. In black **(r)**, are represented all the women from the non-infected group; in red (1), those who had newborns below the 10^th^ percentile; and in blue (0), the women who had newborns within the adequate range for their gestational age. The total number of observations for the non-infected group were n = 124 and *P. vivax* n = 112. *p < 0.05; **p < 0.01; ***p < 0.0001. Abbreviations: HC, head circumference; Ang, angiopoietin; Tie, tyrosine kinase; VEGF, vascular endothelial growth factor; sFlt1, receptor 1 of VEGF; R2GF, receptor 2 of VEGF; PlGF, placental growth factor; sENG, soluble endoglin.

**Fig 4:**
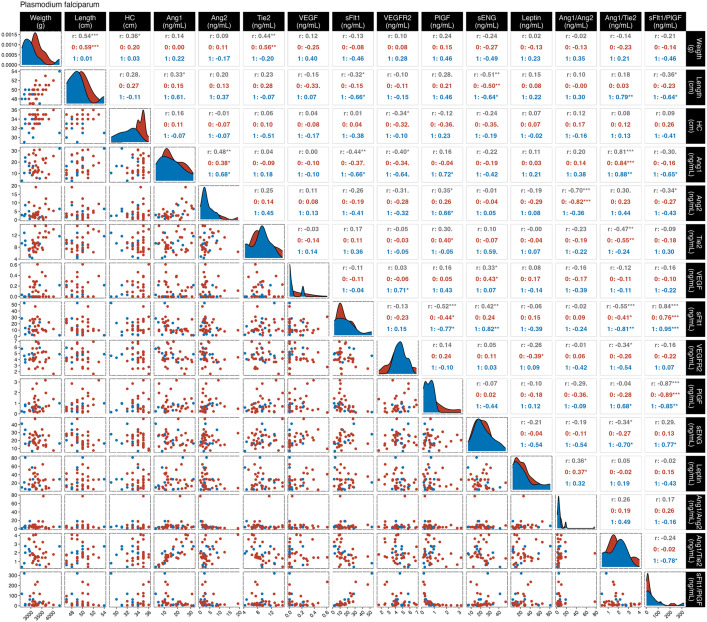
Correlation between maternal peripheral proteins with newborn growth status in the *P. falciparum*-infected group. In black **(r)**, are represented all the women from the non-infected group; in red (1), those who had newborns below the 10^th^ percentile; and in blue (0), the women who had newborns within the adequate range for their gestational age. The total number of observations for the non-infected group were n = 124 and *P. falciparum* n = 39. *p < 0.05; **p < 0.01; ***p < 0.0001. Abbreviations: HC, head circumference; Ang, angiopoietin; Tie, tyrosine kinase; VEGF, vascular endothelial growth factor; sFlt1, receptor 1 of VEGF; R2GF, receptor 2 of VEGF; PlGF, placental growth factor; sENG, soluble endoglin.

To further assess the relationships between maternal markers and newborn anthropometric characteristics, a correlation analysis was performed according to infection status (S1 Fig and Fig 3 and 4). In *P. vivax-*infected women (Fig 3), sFlt1 level and sFlt1/PlGF ratio were weakly negative correlated with newborn weight (rho: -0.22, *p* = 0.02; and rho: -0.21, *p* = 0.02, respectively). sFlt1 were also negatively correlated with newborn length (rho: -0.19, *p* = 0.046). Maternal Ang-2 levels correlates negatively with newborn head circumference (rho: -0.25, *p* = 0.01), and a moderate correlation was observed in the women with newborn below the 10^th^ percentile (rho: -0.51, *p *= 0.006). Additionally, sENG levels was negatively correlated with newborn length (rho: -0.41, *p* = 0.03). *P. falciparum-*infected women (Fig 4) had a moderate positive correlation between Tie2 levels and newborn weight (rho: 0.44, *p* = 0.005). In the same group, newborn length correlated positively with Ang-1 (rho: 0.33, *p* = 0.04) and negatively with sFlt1 (rho: -0.32, *p* = 0.049), sENG (rho: -0.51, *p* = 0.001) and sFlt1/PlGF ratio (rho: -0.36, *p* = 0.02). In *P. falciparum-*infected women with neonates below the 10^th^ percentile showed stronger positive correlations of newborn length with Ang-1/Tie2 ratio (rho: 0.79, *p* = 0.006), and negatively for sFlt1 (rho: -0.66, *p* = 0.037), sENG (rho: -0.64, *p* = 0.047), and sFlt1/PlGF ratio (rho: -0.64, *p* = 0.047).

### Peripheral proteins in the classification of outcomes

To better understand the association of these proteins with the growth parameters of newborns, a classification analysis using ROC curves was performed. First, the performance of the proteins in classifying the outcomes was evaluated (S3 Fig, S4 Fig, and S5 Fig). Subsequently, the maximum potential of each biomarker in infected women was evaluated according to the outcome (S3 Table, S4 Table, S5 Table, and S6 Table).

In the *P. falciparum* group, a sFlt1/PlGF ratio cut-off of 52.94 had an acceptable AUC of 71% but low sensitivity (Sens: 57%; Spec: 86%) for head circumference below the 10^th^ percentile (S6 Table). In contrast to the infected groups, the non-infected group showed a higher performance of proteins with an Ang-1/Tie-2 ratio at a cut-off of 2.24 with an acceptable AUC of 70% but low specificity (Sens: 81%; Spec: 59%), and the sFlt1/PlGF ratio with a cut-off of 81.41 presented an AUC of 71% but low sensitivity (Sens: 62%; Spec: 80%) for SGA (S4 Table); additionally, the sENG level had an AUC of 75% with an increase to 76% at the cut-off of 26.01 ng/mL (Sens: 75%; Spec: 78%) for classifying a newborn length below the 10^th^ percentile (S3 Fig and S5 Table). No proteins were associated with outcomes in the *P. vivax* group. Overall, the infection models did not seem to respond to the outcomes and did not show strong associations.

**Fig 5:**
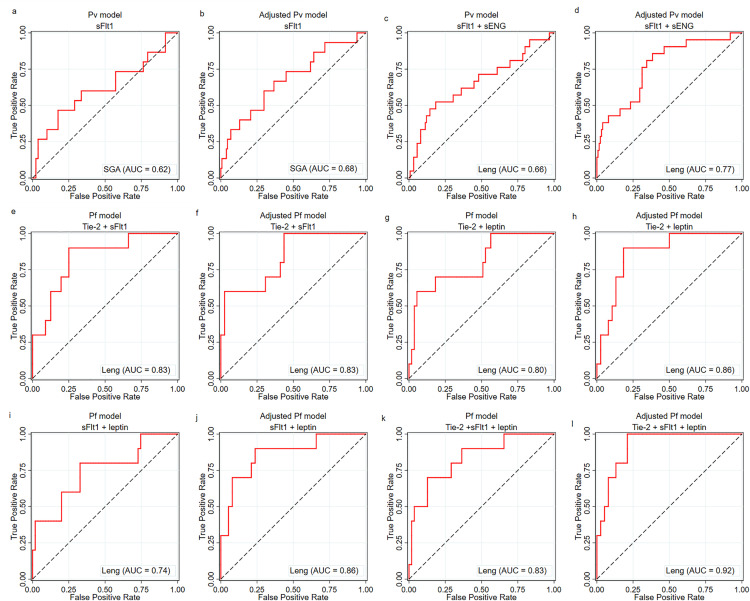
Logistic ROC curves of the delivery maternal peripheral proteins for newborn outcomes, according to species of infection. Abbreviations: *Pv*, *P. vivax*; *Pf*, *P. falciparum*; SGA, small for gestational age; Leng, length; AUC, area under the curve; Tie2, tyrosine kinase; sFlt1, soluble receptor 1 of VEGF; sENG, soluble endoglin.

Since some sociodemographic and maternal clinical data are important in the context of malaria outcomes, the associations of maternal proteins with adverse outcomes were investigated in logistic regression models with adjustment for maternal age bellow 18 years old, education level below 4 years, rural residence, primigravidae, and gestational age (Table 2). Based on this analysis, we selected the proteins with statistical significance for a combination ROC analysis with and without adjustment for the same cofounding variables previous mentioned (Fig 5).

**Table 2:** Association of maternal peripheral proteins with newborn anthropometric outcomes.

	Non-infected^a^				*P. vivax*				*P. falciparum*			
	OR (95% IC)	*p*	aOR (95% IC)	*p*	OR (95% IC)	*p*	aOR (95% IC)	*p*	OR (95% IC)	*p*	aOR (95% IC)	*p*
Ang-1												
SGA ^b^	1.00 (0.93-1.07)	0.92	0.99 (0.92-1.07)	0.84	1.01 (0.96-1.07)	0.69	1.01 (0.96-1.07)	0.68	0.92 (0.81-1.06)	0.26	0.90 (0.77-1.04)	0.16
Length <10^th^	1.01 (0.93-1.09)	0.88	1.01 (0.93-1.10)	0.77	1.00 (0.95-1.05)	0.97	1.01 (0.95-1.06)	0.86	0.96 (0.87-1.06)	0.45	0.93 (0.83-1.05)	0.25
HC < 10^th^	1.02 (0.94-1.09)	0.68	1.02 (0.94-1.10)	0.60	1.04 (0.99-1.08)	0.14	1.04 (0.99-1.09)	0.12	0.99 (0.94-1.05)	0.78	0.99 (0.91-1.06)	0.73
Ang-2												
SGA	0.77 (0.53-1.13)	0.19	0.77 (0.53-1.11)	0.16	0.73 (0.49-1.06)	0.10	0.73 (0.49-1.10)	0.13	0.92 (0.69-1.22)	0.57	0.92 (0.70-1.22)	0.58
Length <10^th^	0.95 (0.73-1.23)	0.68	0.89 (0.66-1.19)	0.43	0.94 (0.78-1.13)	0.51	0.90 (0.73-1.12)	0.34	0.76 (0.52-1.12)	0.17	0.76 (0.51-1.13)	0.18
HC < 10^th^	0.97 (0.76-1.24)	0.83	0.98 (0.76-1.26)	0.89	1.15 (1.02-1.29)	0.03	1.13 (0.99-1.30)	0.07	1.00 (0.83-1.20)	1.00	0.99 (0.80-1.21)	0.91
Tie2												
SGA	0.84 (0.68-1.04)	0.12	0.82 (0.66-1.02)	0.08	0.89 (0.75-1.06)	0.19	0.89 (0.75-1.06)	0.20	0.91 (0.67-1.22)	0.53	0.97 (0.70-1.32)	0.83
Length <10^th^	0.93 (0.75-1.16)	0.54	0.90 (0.72-1.13)	0.34	0.99 (0.87-1.12)	0.86	0.97 (0.85-1.10)	0.62	0.62 (0.43-0.90)	**0.01**	0.64 (0.42-0.97)	**0.04**
HC < 10^th^	1.02 (0.84-1.25)	0.82	1.00 (0.80-1.24)	0.98	1.05 (0.93-1.19)	0.42	1.02 (0.90-1.16)	0.73	1.06 (0.84-1.33)	0.62	1.35 (0.97-1.88)	0.07
VEGF												
SGA	3.58 (0.05-234.60)	0.55	4.22 (0.05-332.90)	0.52	0.98 (0.18-5.39)	0.98	0.95 (0.18-4.93)	0.95	0.01 (<0.01-60.37)	0.28	0.00 (<0.01-106.65)	0.28
Length <10^th^	6.53 (0.07-643.55)	0.42	16.14 (0.10- > 500)	0.28	2.75 (0.67-11.22)	0.16	2.16 (0.51-9.15)	0.30	0.04 (0.00-8.34)	0.23	0.08 (0.00-54.66)	0.44
HC < 10^th^	0.55 (0.00-118.36)	0.83	0.93 (0.00-305.40)	0.98	0.73 (0.09-6.23)	0.77	0.63 (0.08-4.86)	0.66	1.71 (0.03-110.04)	0.80	2.57 (0.01-571.20)	0.73
sFlt1												
SGA	1.03 (0.99-1.07)	0.16	1.03 (0.99-1.07)	0.17	1.03 (1.00-1.07)	**0.04**	1.05 (1.01-1.09)	**0.03**	1.01 (0.94-1.07)	0.82	1.01 (0.94-1.09)	0.70
Length <10^th^	0.99 (0.94-1.04)	0.71	0.99 (0.94-1.05)	0.75	1.04 (1.01-1.07)	**0.02**	1.04 (1.01-1.08)	**0.01**	0.90 (0.81-1.00)	0.05	0.85 (0.72-0.99)	**0.03**
HC < 10^th^	0.98 (0.93-1.04)	0.55	0.98 (0.93-1.03)	0.46	1.02 (0.99-1.05)	0.21	1.01 (0.98-1.04)	0.48	1.02 (0.97-1.08)	0.41	1.02 (0.94-1.10)	0.65
VEGFR2												
SGA	1.43 (0.97-2.11)	0.07	1.47 (0.97-2.22)	0.07	1.23 (0.84-1.81)	0.28	1.23 (0.84-1.81)	0.28	0.80 (0.42-1.54)	0.51	0.85 (0.43-1.68)	0.64
Length <10^th^	0.64 (0.38-1.07)	0.09	0.58 (0.32-1.06)	0.08	0.99 (0.71-1.39)	0.96	0.97 (0.69-1.37)	0.88	0.69 (0.39-1.23)	0.21	0.70 (0.38-1.28)	0.24
HC < 10^th^	0.99 (0.62-1.58)	0.97	1.05 (0.62-1.77)	0.87	1.26 (0.89-1.78)	0.20	1.27 (0.89-1.80)	0.19	0.77 (0.45-1.33)	0.36	1.16 (0.58-2.34)	0.67
PlGF												
SGA	0.67 (0.20-2.21)	0.51	0.66 (0.19-2.27)	0.51	1.21 (0.53-2.73)	0.65	1.13 (0.49-2.61)	0.77	0.37 (0.06-2.43)	0.30	0.30 (0.04-2.48)	0.26
Length <10^th^	1.06 (0.34-3.30)	0.92	1.08 (0.31-3.74)	0.91	0.76 (0.31-1.88)	0.56	0.67 (0.24-1.84)	0.43	0.22 (0.03-1.53)	0.13	0.11 (0.01-1.10)	0.06
HC < 10^th^	0.42 (0.08-2.15)	0.30	0.38 (0.06-2.27)	0.29	1.12 (0.52-2.42)	0.77	1.16 (0.50-2.69)	0.72	0.36 (0.08-1.65)	0.19	0.34 (0.04-2.90)	0.32
Endoglin												
SGA	1.02 (0.98-1.07)	0.32	1.01 (0.97-1.06)	0.59	1.02 (0.98-1.06)	0.41	1.04 (0.99-1.09)	0.15	1.04 (0.99-1.09)	0.12	1.09 (1.00-1.18)	0.05
Length <10^th^	1.07 (1.02-1.12)	**0.003**	1.06 (1.01-1.12)	**0.02**	1.04 (1.00-1.08)	**0.03**	1.06 (1.01-1.11)	**0.01**	1.01 (0.96-1.06)	0.83	1.04 (0.97-1.12)	0.27
HC < 10^th^	1.04 (1.00-1.09)	0.06	1.03 (0.98-1.08)	0.27	1.03 (1.00-1.07)	0.08	1.03 (0.98-1.07)	0.25	1.02 (0.97-1.06)	0.46	1.07 (0.99-1.16)	0.08
Leptin												
SGA	0.99 (0.97-1.02)	0.62	0.99 (0.96-1.02)	0.49	1.01 (0.99-1.03)	0.50	1.01 (0.99-1.03)	0.44	1.00 (0.96-1.05)	0.84	1.00 (0.96-1.05)	0.99
Length <10^th^	1.00 (0.98-1.03)	0.83	1.00 (0.97-1.03)	0.90	1.01 (0.99-1.03)	0.54	1.01 (0.99-1.03)	0.33	1.03 (1.00-1.07)	0.05	1.05 (1.00-1.11)	**0.04**
HC < 10^th^	1.00 (0.97-1.03)	0.90	0.99 (0.96-1.03)	0.71	0.99 (0.96-1.01)	0.36	0.99 (0.96-1.01)	0.40	1.00 (0.96-1.03)	0.90	1.01 (0.96-1.06)	0.72
Ang-1/Ang-2												
SGA	1.01 (1.00-1.02)	0.20	1.01 (1.00-1.02)	0.16	1.01 (0.97-1.04)	0.68	1.01 (0.97-1.04)	0.78	0.98 (0.89-1.07)	0.64	0.97 (0.89-1.07)	0.57
Length <10^th^	0.99 (0.96-1.03)	0.66	1.00 (0.97-1.03)	0.88	1.00 (0.97-1.03)	0.98	1.01 (0.98-1.05)	0.48	1.00 (0.96-1.04)	0.96	0.99 (0.95-1.04)	0.81
HC < 10^th^	0.99 (0.94-1.03)	0.51	0.99 (0.95-1.03)	0.62	0.98 (0.94-1.02)	0.37	0.99 (0.94-1.04)	0.70	0.98 (0.90-1.06)	0.55	0.93 (0.82-1.05)	0.25
Ang-1/Tie2												
SGA	1.01 (0.84-1.22)	0.90	1.02 (0.84-1.24)	0.82	1.15 (0.79-1.68)	0.46	1.16 (0.78-1.72)	0.48	0.72 (0.30-1.71)	0.45	0.54 (0.18-1.58)	0.26
Length <10^th^	0.99 (0.77-1.26)	0.92	1.02 (0.80-1.31)	0.87	0.98 (0.67-1.42)	0.91	1.04 (0.70-1.55)	0.85	1.08 (0.73-1.61)	0.70	0.96 (0.60-1.54)	0.86
HC < 10^th^	0.99 (0.77-1.26)	0.91	1.02 (0.79-1.31)	0.86	1.20 (0.86-1.68)	0.29	1.32 (0.91-1.90)	0.15	0.88 (0.51-1.53)	0.65	0.55 (0.21-1.43)	0.22
sFlt1/PlGF												
SGA	1.00 (1.00-1.00)	0.46	1.00 (1.00-1.00)	0.49	1.00 (1.00-1.00)	0.25	1.00 (1.00-1.00)	0.47	1.00 (0.99-1.02)	0.86	1.02 (0.99-1.06)	0.18
Length <10^th^	1.00 (1.00-1.00)	0.65	1.00 (1.00-1.00)	0.71	1.00 (1.00-1.00)	0.29	1.00 (1.00-1.00)	0.54	0.99 (0.97-1.02)	0.60	0.99 (0.95-1.02)	0.44
HC < 10^th^	1.00 (1.00-1.00)	0.55	1.00 (1.00-1.00)	0.68	1.00 (1.00-1.00)	0.18	1.00 (1.00-1.00)	0.24	1.01 (1.00-1.02)	0.14	1.01 (0.99-103)	0.29

^a^Percentile based on the *Intergrowth* 21st. The units of measurement for the proteins are ng/mL. Abbreviations: NI, non-infected; *Pv*, *Plasmodium vivax*; *Pf*, *Plasmodium falciparum*; SGA, small for gestational age; OR, Odds ratio; aOR, adjusted Odds ratio; HC, head circumference; Ang, angiopoietin; Tie, tyrosine kinase; VEGF, vascular endothelial growth factor; sFlt1, receptor 1 of VEGF; VEGFR2, receptor 2 of VEGF; PlGF, placental growth factor; sENG, soluble endoglin. Each model was adjusted for maternal age (<18 years old), education (<4 years), rural residence, gravidity (primigravidae), and gestational age.

In the *P. vivax* group, the sFlt1 level was associated with reduced newborn weight (Table 2), but there was no power of classification in the unadjusted or adjusted models (Fig 5A-B). However, the sFlt1 and sENG levels were associated with neonatal length (Table 2), and in combination, these two proteins presented an acceptable power of classification in the adjusted model but low sensitivity (AUC: 77%, Sens: 19.1% and Spec: 98.4%) (Fig 5D). Finally, the *P. falciparum*-infected group presented three proteins related to newborn length reduction: Tie2, sFlt1, and leptin (Table 2). These proteins, in combination, had good AUCs in all the models (Fig 5E-L). Using the three biomarkers in an unadjusted model, an AUC of 83% was obtained, with a sensitivity of 40% and a specificity of 98.2% (Fig 5K), and after adjusting for confounding variables, there was an increase in the AUC but a decrease in the sensitivity (AUC: 93%, Sens: 30%, Spec: 97.4%) (Fig 5L).

## Discussion

**Fig 6:**
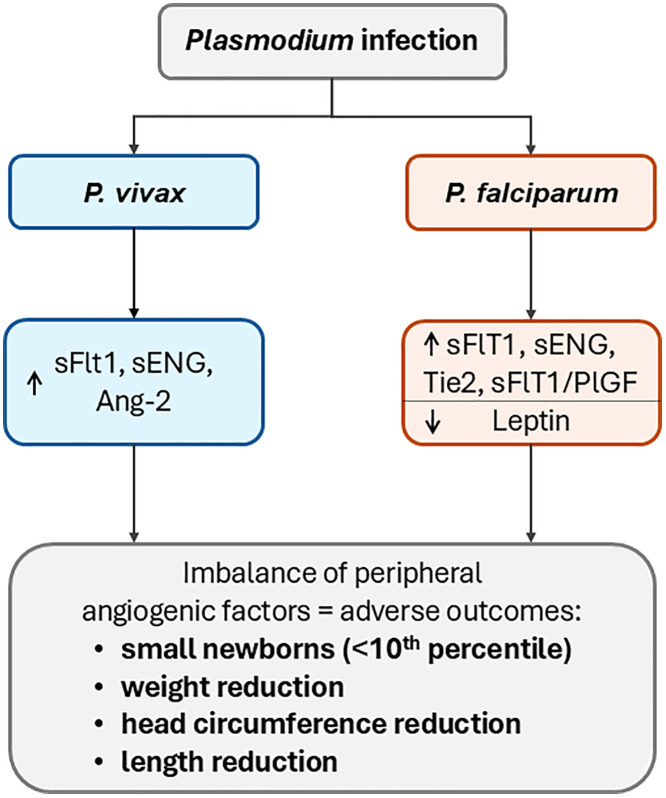
Flowchart of main results.

Our study evaluated the performance of nine maternal peripheral markers at delivery in women from a malaria-endemic region of Brazil and associate these markers with newborn anthropometric characteristics in the context of MiP. We found that maternal Tie2, sFlt1, sENG and leptin levels were associated with a reduction in the anthropometric measurements of newborns (Fig 6). The *P. vivax*-infected group presented an association between peripheral sFlt1 levels and newborn weight and length, and the sENG level was associated with newborn length. In *P. falciparum*-infected women, associations of Tie2, sFlt1, and leptin levels with newborn length were previously observed [23,25,28].

Angiogenesis factors such as Ang-1, Ang-2, VEGF, and sFlt-1 are important for placental growth and vascularisation [10,22], and alterations in these factors can affect placental and foetal development [22]. *Plasmodium* spp. infection before 24 weeks of gestation were associated with alterations of some proteins such as leptin and sENG [29]. In infected primigravidae, angiopoietin dysregulation at delivery was associated with placental malaria and low birth weight, with an increased Ang-2/Ang-1 ratio compared with non-infected women with normal-weight babies [28]. Reduced Ang-1 levels in pregnant women were also associated with histopathological placental changes [23]. In fact, growth restriction in the context of malaria has been associated with the severity of placental damage in *P. falciparum*-infected women [22].

*P. falciparum*-infected women presented greater changes in protein levels than non-infected women did, and both *P. vivax-* and *P. falciparum*-infected women presented similar protein profiles, with an increase in the Tie2 level and a decrease in the Ang-1/Tie2 ratio. For *P. vivax*-infected women, increased Ang-2 levels were correlated with lower neonatal cephalic perimeter, increased sFlt1 levels were associated with low newborn weight and length, and increased sENG levels were associated with associated with reduced newborn length in an adjusted model. There is evidence that *P. vivax* infection may increase risk of maternal anaemia, prematurity, low birth weight, and reduced head circumference [8]. *P. vivax* infection may share several features common to *P. falciparum* infection, such as monocyte infiltration and changes in placental Ang-2 and C5a levels [8].

Peripheral sFlt1 levels are reportedly decreased in women with placental malaria [30]. Under normal physiological conditions, circulating levels of sFlt1 increase throughout pregnancy, particularly in the third trimester, and in women with preeclampsia, there is a greater increase in this protein and in sENG levels early in pregnancy [31]. A different profile was observed in the context of MiP, with a decrease in the plasma levels of sFlt1 and, consequently, a decrease in the sFlt1/PlGF ratio. Nevertheless, the correlation analysis revealed that the sFlt1 level, and sFlt1/PlGF ratio were negatively related to neonatal weight and that the sFlt1 level was negatively related to neonatal length, indicating that higher levels of these proteins are associated with lower anthropometric measurements in newborns. Therefore, this correlation is similar to women with preeclampsia, where increases in sFlt1 and sENG are associated with FGR [31]. In *P. falciparum*-infected women, there was also a decrease in leptin levels and the Ang-1/Ang-2 ratio and associations among Tie2, sFlt1, and leptin levels with reduced newborn length. Leptin levels are expected to increase during pregnancy, peaking in the third trimester [32], but a decrease in this protein has previously been associated with FGR and occult placental malaria [30].

The individual performance of each biomarker in *P. falciparum*-infected women revealed that a cut-off of 81.41 for the sFlt1/PlGF ratio had an acceptable performance (AUC: 71%) but low sensitivity (57%) for classifying head circumference below the 10^th^ percentile. Other studies have associated a high sFlt1/PlGF ratio with the severity of early-onset FGR [33], and a cut-off value of 38 for this ratio was predictive of early delivery [34]. This ratio was also increased in pregnancies complicated by preeclampsia and FGR [35]. None of the proteins were suitable for classifying small newborns in the *P. vivax* group. However, for non-infected women, sENG at a cut-off of 26.01 ng/mL performed well for classifying newborn length in the non-infected group (AUC: 76%, Sens: 75%, Spec: 78%). This could indicate that altered sENG might be associated with newborn size regardless of maternal infection status.

Studies have shown that biomarkers used together with other clinical parameters may have better performance to access growth parameters [31]. Based on this, we tested the classification power of potential markers for negative neonatal outcomes. Maternal Tie2, sFlt1, and leptin levels presented good power of classification for newborn lengths below the 10^th^ percentile, but these markers had low sensitivity in *P. falciparum*-infected women.

One study performed in Poland showed that serum sENG levels were increased not only in women with preeclampsia but also in normotensive women with newborns below the 10^th^ percentile in comparison with those of women in the control group [36]. Low PlGF and high sFlt1/PlGF ratio maternal levels were observed in Spanish women which delivered newborns with congenital heart defect [37]. In the same study, higher sFlt1 and sFlt1/PlGF ratio associated with reduced birth weight and head circumference [37]. Similar findings in Zambia have shown that mid-trimester proteins, specifically high PlGF and sFlt1 and low sENG were associated with small for gestational age newborns [38].

The results presented here contribute to a better understanding of the imbalance of these molecules in women with MiP, which are associated with neonatal adverse outcomes. However, this study has some limitations that may affect the interpretability and generalizability of the results. First, the samples used here were collected from a specific area in Acre state. Also, as the newborns were not followed up after birth, it was not possible to access catch-up growth status, as there is evidence that babies with a birth weight less than the 10^th^ percentile might be born small but healthy [10]. To minimize this limitation, in addition to newborn weight, we also considered in our study other anthropometric measures such as length and head circumference. Additionally, some ELISA kits have a limited range of detection, resulting in undetectable values. Another limitation is the failure to assess other variables that are important for foetal growth, such as the number of infections and the gestational period in which these infections occurred, as well as the nutritional status of the pregnant woman; these variables were not assessed in this study due to lack of information and the overall objective of the study, which was to assess the population of pregnant women as a whole at the end of pregnancy, regardless of the difference in these criteria. Finally, the stratification of some variables led to an imbalance in the data when one variable had a greater number of observations than the other, limiting our sample size and statistical power of some analyses.

Knowing that placental malaria is relevant to foetal development and various gestational consequences, information on placental analysis can be found in two other studies that analysed this cohort of pregnant women with this objective [8,23,25]. The current study is exploratory, and future studies should be conducted to experimentally validate the involvement of the identified factors and the underlying mechanisms.

In conclusion, our results provide insights that an imbalance in maternal peripheral protein levels might be important in identifying babies at risk of growth imbalance. This suggests that these proteins could be used to identify babies at risk of malaria-associated growth imbalance, but more studies would be needed to confirm this finding. Using peripheral proteins to access health status could benefit pregnant women in malaria endemic regions. More studies should be conducted to understand the relationship between changes in these peripheral protein levels and changes in the placenta so that we can obtain a more accurate estimation of the insults caused by MiP.

## Supporting information

S1 STROBE Statement Checklist of items that should be included in reports of cross-sectional studies. (DOCX)

S1 Table Enzyme-Linked Immunosorbent Assay (ELISA) protocols details. (DOCX)

S2 Table Peripheral maternal protein levels at delivery. (DOCX)

S3 Table Youden index cut-off points of maternal peripheral proteins for newborn size below the 10th percentile. (DOCX)

S4 Table Youden index cut-off points of maternal peripheral proteins for weight below the 10th percentile. (DOCX)

S5 Table Youden index cut-off points of maternal peripheral proteins for length below 10th percentile. (DOCX)

S6 Table Youden index cut-off points of maternal peripheral proteins for head circumference below the 10th percentile. (DOCX)

S1 Fig Flow chart of selection of pregnant women in the study. (DOCX)

S2 Fig Correlation between maternal peripheral proteins with newborn growth status in the non-infected group. (DOCX)

S3 Fig ROC curve performance of maternal peripheral proteins for newborn growth status in the non-infected group. (DOCX)

S4 Fig ROC curve performance of maternal peripheral proteins for newborn growth status in the P. vivax-infected group. (DOCX)

S5 Fig ROC curve performance of maternal peripheral proteins for newborn growth status in the P. falciparum-infected group. (DOCX)
