# Supplementary material for: Association between peripheral markers in women with malaria in pregnancy and small newborns: A cross-sectional study
Source: PLOS Glob Public Health. 2025 Dec 3;5(12):e0005526. doi: 10.1371/journal.pgph.0005526 (PMC12674551; doi:10.1371/journal.pgph.0005526)
Supplement: S1 STROBE Statement — (DOCX) [file pgph.0005526.s001.docx]

**S1 STROBE Statement. Checklist of items that should be included in reports of cross-sectional studies**

|  | Item No | Recommendation | Page | Relevant text from the manuscript |
| --- | --- | --- | --- | --- |
| **Title and abstract** | 1 | (*a*) Indicate the study’s design with a commonly used term in the title or the abstract | 1 | A cross-sectional study |
|  |  | (*b*) Provide in the abstract an informative and balanced summary of what was done and what was found | 2 | See **Summary** |
| Introduction | | |  |  |
| Background/rationale | 2 | Explain the scientific background and rationale for the investigation being reported | 3, 4 | See **Introduction** |
| Objectives | 3 | State specific objectives, including any prespecified hypotheses | 4 | Maternal peripheral angiogenic and proangiogenic proteins may play a key role in understanding FGR associated with MiP. Therefore, we intend to identify potential maternal peripheral proteins and their association with weight, length, and head reduction in newborns in the context of MiP. |
| Methods | | |  |  |
| Study design | 4 | Present key elements of study design early in the paper | 4, 5 | See **Study site and population** in Method’s section. |
| Setting | 5 | Describe the setting, locations, and relevant dates, including periods of recruitment, exposure, follow-up, and data collection | 4, 5 | See **Study site and population** in Method’s section. |
| Participants | 6 | (*a*) Give the eligibility criteria, and the sources and methods of selection of participants | 4, 5 | See **Study site and population** in Method’s section. |
| Variables | 7 | Clearly define all outcomes, exposures, predictors, potential confounders, and effect modifiers. Give diagnostic criteria, if applicable | 4-6 | See **Study site and population, Anthropometric measurements, and Protein measurements** in Method’s section. |
| Data sources/ measurement | 8 | For each variable of interest, give sources of data and details of methods of assessment (measurement). Describe comparability of assessment methods if there is more than one group | 4-6 | See **Study site and population, Anthropometric measurements, and Protein measurements** in Method’s section. |
| Bias | 9 | Describe any efforts to address potential sources of bias- | 5-6 | See **Anthropometric measurements, Protein measurements, and Statistical analysis** in Method’s section. |
| Study size | 10 | Explain how the study size was arrived at | 4-5 | See **Study site and population** in Method’s section. |
| Quantitative variables | 11 | Explain how quantitative variables were handled in the analyses. If applicable, describe which groupings were chosen and why | 6 | See **Statistical analysis** in Method’s section. |
| Statistical methods | 12 | (*a*) Describe all statistical methods, including those used to control for confounding | 6 | See **Statistical analysis** in Method’s section. |
|  |  | (*b*) Describe any methods used to examine subgroups and interactions | 6 | See **Statistical analysis** in Method’s section. |
|  |  | (*c*) Explain how missing data were addressed | 6 | See **Statistical analysis** in Method’s section. |
|  |  | (*d*) If applicable, describe analytical methods taking account of sampling strategy | - | N.A. |
|  |  | (*e*) Describe any sensitivity analyses | - | N.A. |
| Results | | |  |  |
| Participants | 13 | (a) Report numbers of individuals at each stage of study—eg numbers potentially eligible, examined for eligibility, confirmed eligible, included in the study, completing follow-up, and analysed | 5-7 | See **Figure 1** in **Methods** and **Study population and baseline characteristics** in **Results’** section. |
|  |  | (b) Give reasons for non-participation at each stage | 5-7 | See **Figure 1** in **Methods** and **Study population and baseline characteristics** in **Results’** section. |
|  |  | (c) Consider use of a flow diagram | 5 | See **Figure 1** in **Methods.** |
| Descriptive data | 14 | (a) Give characteristics of study participants (eg demographic, clinical, social) and information on exposures and potential confounders | 7 | See **Study population and baseline characteristics,** and **Table 1** in **Results’** section. |
|  |  | (b) Indicate number of participants with missing data for each variable of interest | 8 | See **Study population and baseline characteristics** and footnotes of each Table and Figures in **Results’** section. |
| Outcome data | 15 | Report numbers of outcome events or summary measures | 7-10 | See the **Results’** section and **Supplementary** material. |
| Main results | 16 | (*a*) Give unadjusted estimates and, if applicable, confounder-adjusted estimates and their precision (eg, 95% confidence interval). Make clear which confounders were adjusted for and why they were included | 7-10 | See **Results.** |
|  |  | (*b*) Report category boundaries when continuous variables were categorized | - | N.A. |
|  |  | (*c*) If relevant, consider translating estimates of relative risk into absolute risk for a meaningful time period | - | N.A. |
| Other analyses | 17 | Report other analyses done—eg analyses of subgroups and interactions, and sensitivity analyses | - | N.A. |
| Discussion | | |  |  |
| Key results | 18 | Summarise key results with reference to study objectives | 10-11 | See first paragraph in **Discussion’** section**.** |
| Limitations | 19 | Discuss limitations of the study, taking into account sources of potential bias or imprecision. Discuss both direction and magnitude of any potential bias | 13 | See the penultimate paragraph in the **Discussion’** section. |
| Interpretation | 20 | Give a cautious overall interpretation of results considering objectives, limitations, multiplicity of analyses, results from similar studies, and other relevant evidence | 10-13 | See **Discussion.** |
| Generalisability | 21 | Discuss the generalisability (external validity) of the study results | 13 | See the penultimate paragraph in the **Discussion’** section. |
| Other information | | |  |  |
| Funding | 22 | Give the source of funding and the role of the funders for the present study and, if applicable, for the original study on which the present article is based | 2, 14 | See **Funding** in the **Summary** and **Acknowledgements**’ sections. |
