# Supplementary material for: Association between peripheral markers in women with malaria in pregnancy and small newborns: A cross-sectional study
Source: PLOS Glob Public Health. 2025 Dec 3;5(12):e0005526. doi: 10.1371/journal.pgph.0005526 (PMC12674551; doi:10.1371/journal.pgph.0005526)
Supplement: S1 Table — (DOCX) [file pgph.0005526.s002.docx]

**S1 Table. Enzyme-Linked Immunosorbent Assay (ELISA) protocols details.**

| **Protein** | **Company** | **Dilution** | **No. samples** | **Detection range** | **Protocol changes** |
| --- | --- | --- | --- | --- | --- |
| **Ang-1** | R&D* | 1:20 | 385 | 156 – 10000 pg/mL | 50µl/well |
| **Ang-2** | R&D | 1:10 | 386 | 93.8 – 6000 pg-mL | 50µl/well |
| **Tie-2** | R&D | 1:20 | 386 | 156 – 10000 pg/mL | 50µl/well |
| **VEGF** | R&D | 1:10 | 379 | 31.3 – 2000 pg/mL | 5 times wash, 50µl/well |
| **sFlt1** | R&D | 1:5 | 386 | 125 – 8000 pg/mL | Samples at 50µl/well |
| **VEGFR2** | R&D | 1:10 | 385 | 250 - 4000 pg/mL | 50µl/well |
| **PlGF** | R&D | 1:5 | 386 | 31.3 – 2000 pg/mL | 50µl/well |
| **sENG** | R&D | 1:50 | 386 | 125 – 8000 pg/mL | 5 times wash, 50µl/well |
| **Leptin** | R&D | 1:50 | 384 | 31.2 – 2000 pg/mL | 50µl/well |

Abbreviations: Ang, angiopoietin; Tie, tyrosine kinase; VEGF, vascular endothelial growth factor; sFlt1, soluble receptor 1 of VEGF; PlGF, placental growth factor; VEGFR2, soluble receptor 2 of VEGF; sENG, soluble endoglin. *R&D Systems (<https://www.rndsystems.com>).
