# Supplementary material for: Association between peripheral markers in women with malaria in pregnancy and small newborns: A cross-sectional study
Source: PLOS Glob Public Health. 2025 Dec 3;5(12):e0005526. doi: 10.1371/journal.pgph.0005526 (PMC12674551; doi:10.1371/journal.pgph.0005526)
Supplement: S2 Table — (DOCX) [file pgph.0005526.s003.docx]

**S2 Table. Peripheral maternal protein levels at delivery.**

| **Protein, ng/mL** | **NI total**  (n = 128-169) | ***Pv* total**  (n = 117-150) | ***Pf* total**  (n = 42-67) | **NI AGA** | ***Pv* AGA** | ***Pf* AGA** | **NI <10^th^** | ***Pv* <10^th^** | ***Pf* <10^th^** |
| --- | --- | --- | --- | --- | --- | --- | --- | --- | --- |
| **Ang-1** | 14.74  [10.80-19.88] | 13.34  [9.91-18.10] | 12.96  [9.44-20.48] | 14.87  [10.31-19.88] | 13.44  [9.89-17.64] | 12.92  [9.92-20.57] | 13.31  [11.52-19.68] | 13.01  [10.38-19.70] | 12.46  [8.92-18.02] |
| **Ang-2** | 1.71  [0.87-2.81] | 1.85  [0.88-3.48] | 2.20  [1.20-4.30] | 1.76  [0.95-3.02] | 1.97  [0.89-3.45] | 2.44  [1.33-4.30] | 1.30  [0.65-2.52] | 1.63  [0.95-3.85] | 2.18  [0.90-4.22] |
| **Tie-2** | 6.96  [5.59-9.11] | 8.53  [7.03-11.81] | 9.14  [7.03-11.13] | 7.27  [5.71-9.32] | 8.72  [7.02-11.52] | 9.03  [7.37-11.13] | 6.21  [5.49-7.69] | 7.87  [7.06-12.29] | 9.22  [6.78-10.76] |
| **VEGF** | 0.00  [0.00-0.11] | 0.11  [0.00-0.23] | 0.04  [0.00-0.25] | 0.00  [0.00-0.11] | 0.11  [0.00-0.23] | 0.09  [0.00-0.27] | 0.00  [0.00-0.11] | 0.16  [0.00-0.23] | 0.00  [0.00-0.21] |
| **sFlt1** | 14.24  [8.38-23.48] | 14.9  [7.85-26.97] | 10.04  [3.89-21.19] | 14.03  [7.97-21.41] | 14.42  [8.18-23.78] | 10.36  [8.15-22.23] | 19.64  [11.11-28.66] | 19.95  [7.82-40.05] | 6.95  [2.27-18.67] |
| **VEGFR2** | 4.00  [3.16-4.81] | 4.67  [3.73-5.61] | 4.12  [3.56-5.11] | 3.99  [3.13-4.70] | 4.63  [3.60-5.50] | 4.21  [3.67-5.30] | 4.20  [3.41-4.96] | 4.89  [3.98-5.87] | 4.02  [3.20-4.72] |
| **PlGF** | 0.33  [0.05-0.67] | 0.34  [0.10-0.69] | 0.26  [0.00-0.74] | 0.35  [0.05-0.67] | 0.35  [0.11-0.71] | 0.46  [0.00-0.85] | 0.26  [0.07-0.59] | 0.34  [0.02-0.61] | 0.16  [0.00-0.61] |
| **sENG** | 20.46  [14.74-26.35] | 20.75  [16.24-29.91] | 19.65  [14.69-27.09] | 20.03  [14.59-25.91] | 20.64  [16.00-28.40] | 20.45  [14.79-27.68] | 21.18  [16.77-27.36] | 23.13  [16.49-35.75] | 17.35  [12.89-25.16] |
| **Leptin** | 27.01  [16.81-42.77] | 20.90  [11.89-38.63] | 17.62  [9.28-29.51] | 27.19  [15.44-44.54] | 20.90  [12.53-38.83] | 17.99  [11.03-29.73] | 25.53  [19.29-33.71] | 23.92  [9.22-38.53] | 14.08  [6.65-29.28] |
| **Ang-1/Ang-2** | 8.87  [4.51-18.71] | 7.15  [3.50-15.43] | 4.81  [3.32-9.04] | 8.43  [4.27-17.54] | 6.95  [3.45-16.57] | 4.96  [3.71-9.32] | 11.48  [5.06-25.39] | 7.20  [4.88-14.87] | 4.55  [3.29-8.83] |
| **Ang-1/Tie-2** | 2.05  [1.29-3.14] | 1.49  [0.99-2.29] | 1.64  [0.97-2.36] | 1.98  [1.26-3.05] | 1.51  [0.98-2.25] | 1.54  [1.05-2.16] | 2.60  [1.71-3.48] | 1.46  [1.02-2.66] | 1.85  [0.80-2.31] |
| **sFlt1/PlFG** | 39.23  [17.90-81.41] | 38.46  [16.52-79.05] | 14.71  [7.78-41.99] | 35.87  [16.60-66.71] | 37.24  [16.65-68.89] | 13.92  [8.88-40.69] | 61.39 [21.61-149.95] | 43.00  [8.60-109.56] | 15.50  [4.81-63.15] |

Data are presented as median and interquartile range. Abbreviations: NI, non-infected; Pv, *P. vivax*; Pf, *P. falciparum*; AGA, adequate for gestational age; Ang, angiopoietin; Tie, tyrosine kinase; VEGF, vascular endothelial growth factor; sFlt1, soluble receptor 1 of VEGF; PlGF, placental growth factor; VEGFR2, soluble receptor 2 of VEGF; sENG, soluble endoglin.
