# Supplementary material for: Association between peripheral markers in women with malaria in pregnancy and small newborns: A cross-sectional study
Source: PLOS Glob Public Health. 2025 Dec 3;5(12):e0005526. doi: 10.1371/journal.pgph.0005526 (PMC12674551; doi:10.1371/journal.pgph.0005526)
Supplement: S3 Table — (DOCX) [file pgph.0005526.s004.docx]

**S3 Table. Youden index cut-off points of maternal peripheral proteins for newborn size below the 10^th^ percentile.**

| **<10^th^** | **Non-infected group** | | | | **Malaria group** | | | | ***Pv* group** | | | | ***Pf* group** | | | |
| --- | --- | --- | --- | --- | --- | --- | --- | --- | --- | --- | --- | --- | --- | --- | --- | --- |
| **Proteins** | **Cut-off^a^** | **J** | **AUC** | **Sens-Spec** | **Cut-off^a^** | **J** | **AUC** | **Sens-Spec** | **Cut-off^a^** | **J** | **AUC** | **Sens-Spec** | **Cut-off^a^** | **J** | **AUC** | **Sens-Spec** |
| **Ang-1** | 10.11 | 0.11 | 0.55 | 87-24% | 19.13 | 0.06 | 0.53 | 29-78% | 19.09 | 0.11 | 0.55 | 31-80% | 14.22 | 0.07 | 0.53 | 50-57% |
| **Ang-2** | 4.52 | 0.04 | 0.52 | 16-88% | 4.73 | 0.07 | 0.54 | 23-84% | 4.04 | 0.09 | 0.54 | 25-84% | 1.98 | 0.06 | 0.53 | 65-41% |
| **Tie-2** | 3.11 | 0.04 | 0.52 | 100-4% | 12.26 | 0.05 | 0.52 | 23-81% | 12.26 | 0.08 | 0.54 | 28-80% | 8.62 | 0.16 | 0.58 | 70-46% |
| **VEGF** | 0.07 | 0. 07 | 0.57 | 42-72% | 0.19 | 0.09 | 0.54 | 40-68% | 0.19 | 0.15 | 0.57 | 46-69% | 0.50 | 0.01 | 0.51 | 6-96% |
| **sFlt1** | 18.71 | 0.22 | 0.61 | 55-67% | 28.15 | 0.21 | 0.61 | 34-87% | 29.92 | 0.31 | 0.65 | 44-86% | 38.65 | 0.08 | 0.54 | 10-98% |
| **VEGFR2** | 3.88 | 0.14 | 0.57 | 68-47% | 5.43 | 0.06 | 0.53 | 30-75% | 5.43 | 0.16 | 0.58 | 42-74% | 1.78 | 0.02 | 0.51 | 100-2% |
| **PlGF** | 0.02 | 0.07 | 0.53 | 84-23% | 0.16 | 0.00 | 0.50 | 66-34% | 0.17 | 0.06 | 0.53 | 72-34% | NA | 0.00 | 0.50 | 0-100% |
| **sENG** | 14.36 | 0.18 | 0.59 | 94-24% | 35.58 | 0.14 | 0.57 | 21-92% | 35.58 | 0.21 | 0.60 | 28-93% | 68.79 | 0.05 | 0.53 | 5-100% |
| **Leptin** | 15.26 | 0.15 | 0.57 | 90-25% | 3.22 | 0.04 | 0.52 | 100-4% | 34.77 | 0.05 | 0.53 | 36-69% | 56.94 | 0.13 | 0.56 | 15-98% |
| **Ang-1/Ang-2** | 7.57 | 0.15 | 0.58 | 68-47% | 4.40 | 0.07 | 0.54 | 71-37% | 5.21 | 0.14 | 0.57 | 75-39% | 13.06 | 0.08 | 0.54 | 21-87% |
| **Ang-1/Tie-2** | 2.18 | 0.25 | 0.62 | 68-57% | 2.14 | 0.09 | 0.55 | 36-74% | 2.51 | 0.12 | 0.56 | 31-82% | 1.92 | 0.17 | 0.59 | 50-67% |
| **sFlt1/PlGF** | 57.63 | 0.25 | 0.63 | 56-69% | 60.43 | 0.14 | 0.57 | 38-75% | 60.43 | 0.11 | 0.56 | 39-72% | 52.94 | 0.20 | 0.60 | 36-84% |

^a^The values of the proteins are displayed as ng/mL. The proteins were measured in maternal plasma at delivery. Non-infected (n = 165-166), Malaria (n = 156-212), *Pv* (n = 144-146), *Pf* (n = 62-66). Abbreviations: *Pv*, *P. vivax*; *Pf*, *P. falciparum*; J, Youden’s index; AUC, area under the curve; Sens, sensibility; Spec, specificity; Ang, angiopoietin; Tie-2, tyrosine kinase; VEGF, vascular endothelial growth factor; sFlt1, soluble VEGF receptor 1; VEGFR2, soluble VEGF receptor 2; PlGF, placental growth factor; sENG, soluble endoglin.
