# Supplementary material for: Association between peripheral markers in women with malaria in pregnancy and small newborns: A cross-sectional study
Source: PLOS Glob Public Health. 2025 Dec 3;5(12):e0005526. doi: 10.1371/journal.pgph.0005526 (PMC12674551; doi:10.1371/journal.pgph.0005526)
Supplement: S4 Table — (DOCX) [file pgph.0005526.s005.docx]

**S4 Table. Youden index cut-off points of maternal peripheral proteins for weight below the 10^th^ percentile.**

| **SGA** | **Non-infected group** | | | | **Malaria group** | | | | ***Pv* group** | | | | ***Pf* group** | | | |
| --- | --- | --- | --- | --- | --- | --- | --- | --- | --- | --- | --- | --- | --- | --- | --- | --- |
| **Proteins** | **Cut-off^a^** | **J** | **AUC** | **Sens-Spec** | **Cut-off^a^** | **J** | **AUC** | **Sens-Spec** | **Cut-off^a^** | **J** | **AUC** | **Sens-Spec** | **Cut-off^a^** | **J** | **AUC** | **Sens-Spec** |
| **Ang-1** | 12.36 | 0.16 | 0.58 | 75-41% | 8.37 | 0.09 | 0.54 | 86-22% | 10.27 | 0.16 | 0.58 | 87-30% | 8.29 | 0.06 | 0.53 | 86-20% |
| **Ang-2** | 4.90 | 0.03 | 0.51 | 13-90% | 0.57 | 0.03 | 0.52 | 91-12% | 0.66 | 0.04 | 0.52 | 87-17% | 1.98 | 0.12 | 0.56 | 71-41% |
| **Tie-2** | 3.48 | 0.07 | 0.53 | 100-7% | 7.38 | 0.05 | 0.53 | 77-28% | 6.73 | 0.09 | 0.54 | 87-22% | 8.62 | 0.14 | 0.57 | 71-42% |
| **VEGF** | 0.07 | 0.22 | 0.61 | 50-72% | 0.19 | 0.18 | 0.59 | 50-68% | 0.19 | 0.28 | 0.64 | 60-68% | NA | 0.00 | 0.50 | 0-100% |
| **sFlt1** | 19.72 | 0.25 | 0.62 | 56-69% | 28.15 | 0.25 | 0.63 | 41-84% | 31.54 | 0.29 | 0.65 | 47-82% | 13.23 | 0.23 | 0.62 | 57-66% |
| **VEGFR2** | 3.46 | 0.30 | 0.65 | 94-36% | 5.59 | 0.09 | 0.55 | 32-77% | 3.84 | 0.21 | 0.61 | 93-28% | 2.57 | 0.14 | 0.57 | 100-14% |
| **PlGF** | 0.03 | 0.12 | 0.56 | 88-24% | 0.52 | 0.07 | 0.53 | 41-66% | 0.52 | 0.15 | 0.58 | 47-69% | 0.24 | 0.03 | 0.51 | 57-46% |
| **sENG** | 24.50 | 0.14 | 0.57 | 44-70% | 32.63 | 0.22 | 0.61 | 36-85% | 35.75 | 0.17 | 0.58 | 27-90% | 32.26 | 0.34 | 0.67 | 43-92% |
| **Leptin** | 18.36 | 0.25 | 0.62 | 94-31% | 56.94 | 0.12 | 0.56 | 23-89% | 20.24 | 0.15 | 0.57 | 67-48% | 56.94 | 0.25 | 0.63 | 29-97% |
| **Ang-1/Ang-2** | 7.97 | 0.30 | 0.65 | 81-49% | 4.50 | 0.25 | 0.62 | 86-39% | 4.47 | 0.36 | 0.68 | 100-36% | 8.23 | 0.15 | 0.58 | 43-72% |
| **Ang-1/Tie-2** | 2.24 | 0.40 | 0.70 | 81-59% | 1.56 | 0.15 | 0.58 | 64-52% | 1.53 | 0.20 | 0.60 | 67-53% | 1.92 | 0.22 | 0.61 | 57-64% |
| **sFlt1/PlGF** | 81.41 | 0.42 | 0.71 | 62-80% | 60.89 | 0.21 | 0.61 | 47-74% | 61.17 | 0.17 | 0.59 | 45-72% | 52.94 | 0.32 | 0.66 | 50-82% |

^a^The values of the proteins are displayed as ng/mL. The proteins were measured in maternal plasma at delivery. Non-infected (n = 165-166), Malaria (n = 156-212), *Pv* (n = 144-146), *Pf* (n = 62-66). Abbreviations: SGA, small for gestational age; *Pv*, *P. vivax*; *Pf*, *P. falciparum*; J, Youden’s index; AUC, area under the curve; Sens, sensibility; Spec, specificity; Ang, angiopoietin; Tie-2, tyrosine kinase; VEGF, vascular endothelial growth factor; sFlt1, soluble VEGF receptor 1; VEGFR2, soluble VEGF receptor 2; PlGF, placental growth factor; sENG, soluble endoglin; NA, non-applicable.
