# Supplementary material for: Association between peripheral markers in women with malaria in pregnancy and small newborns: A cross-sectional study
Source: PLOS Glob Public Health. 2025 Dec 3;5(12):e0005526. doi: 10.1371/journal.pgph.0005526 (PMC12674551; doi:10.1371/journal.pgph.0005526)
Supplement: S5 Table — (DOCX) [file pgph.0005526.s006.docx]

**S5 Table. Youden index cut-off points of maternal peripheral proteins for length below 10^th^ percentile.**

| **Len <10^th^** | **Non-infected group** | | | | **Malaria group** | | | | ***Pv* group** | | | | ***Pf* group** | | | |
| --- | --- | --- | --- | --- | --- | --- | --- | --- | --- | --- | --- | --- | --- | --- | --- | --- |
| **Proteins** | **Cut-off^a^** | **J** | **AUC** | **Sens-Spec** | **Cut-off^a^** | **J** | **AUC** | **Sens-Spec** | **Cut-off^a^** | **J** | **AUC** | **Sens-Spec** | **Cut-off^a^** | **J** | **AUC** | **Sens-Spec** |
| **Ang-1** | 30.38 | 0.13 | 0.56 | 17-96% | 10.27 | 0.11 | 0.55 | 81-30% | 10.99 | 0.16 | 0.58 | 81-35% | 14.22 | 0.05 | 0.53 | 50-55% |
| **Ang-2** | 1.82 | 0.14 | 0.57 | 58-55% | 0.50 | 0.05 | 0.52 | 94-10% | 0.59 | 0.11 | 0.56 | 95-16% | NA | 0.00 | 0.50 | 0-100% |
| **Tie-2** | 9.83 | 0.09 | 0.55 | 25-84% | 4.27 | 0.02 | 0.51 | 97-5% | 5.20 | 0.12 | 0.56 | 100-12% | NA | 0.00 | 0.50 | 0-100% |
| **VEGF** | 0.07 | 0.21 | 0.60 | 50-71% | 0.22 | 0.11 | 0.55 | 39-72% | 0.22 | 0.17 | 0.58 | 43-74% | NA | 0.00 | 0.50 | 0-100% |
| **sFlt1** | 22.92 | 0.17 | 0.58 | 42-75% | 31.64 | 0.22 | 0.61 | 35-87% | 31.54 | 0.37 | 0.69 | 52-85% | NA | 0.00 | 0.50 | 0-100% |
| **VEGFR2** | 1.55 | 0.01 | 0.51 | 100-1% | 5.81 | 0.04 | 0.52 | 23-81% | 6.52 | 0.07 | 0.54 | 14-93% | 2.02 | 0.04 | 0.52 | 100-4% |
| **PlGF** | 1.04 | 0.17 | 0.59 | 25-92% | 2.54 | 0.01 | 0.51 | 3-98% | 0.17 | 0.04 | 0.52 | 71-33% | NA | 0.00 | 0.50 | 0-100% |
| **sENG** | 26.01 | 0.53 | 0.76 | 75-78% | 32.63 | 0.18 | 0.59 | 32-86% | 32.63 | 0.28 | 0.64 | 43-85% | 68.79 | 0.10 | 0.55 | 10-100% |
| **Leptin** | 10.05 | 0.12 | 0.56 | 100-12% | 32.49 | 0.16 | 0.58 | 45-71% | 20.24 | 0.16 | 0.58 | 67-49% | 56.94 | 0.28 | 0.64 | 30-98% |
| **Ang-1/Ang-2** | 7.57 | 0.12 | 0.56 | 67-45% | 5.21 | 0.17 | 0.58 | 73-44% | 5.21 | 0.19 | 0.60 | 81-38% | 18.14 | 0.26 | 0.63 | 33-93% |
| **Ang-1/Tie-2** | 2.60 | 0.23 | 0.61 | 58-64% | 1.24 | 0.15 | 0.57 | 77-38% | 1.01 | 0.15 | 0.57 | 86-29% | 1.53 | 0.30 | 0.65 | 80-50% |
| **sFlt1/PlGF** | 59.89 | 0.23 | 0.61 | 56-67% | 24.58 | 0.20 | 0.60 | 75-45% | 24.58 | 0.20 | 0.60 | 81-39% | 32.35* | NA | 0.59 | 50-68% |

^a^The values of the proteins are displayed as ng/mL. The proteins were measured in maternal plasma at delivery. Non-infected (n = 165-166), Malaria (n = 156-212), *Pv* (n = 144-146), *Pf* (n = 62-66). Abbreviations: Len, length; *Pv*, *P. vivax*; *Pf*, *P. falciparum*; Youden’s index; AUC, area under the curve; Sens, sensibility; Spec, specificity; Ang, angiopoietin; tie, tyrosine kinase; VEGF, vascular endothelial growth factor; sFlt1, soluble VEGF receptor 1; VEGFR2, soluble VEGF receptor 2; PlGF, placental growth factor; sENG, soluble endoglin; NA, non-applicable. *The estimation presented ties, so the method used for this biomarker was Liu cut-off.
