# Supplementary material for: Association between peripheral markers in women with malaria in pregnancy and small newborns: A cross-sectional study
Source: PLOS Glob Public Health. 2025 Dec 3;5(12):e0005526. doi: 10.1371/journal.pgph.0005526 (PMC12674551; doi:10.1371/journal.pgph.0005526)
Supplement: S6 Table — (DOCX) [file pgph.0005526.s007.docx]

**S6 Table. Youden index cut-off points of maternal peripheral proteins for head circumference below the 10^th^ percentile.**

| **HC <10^th^** | **Non-infected group** | | | | **Malaria group** | | | | ***Pv* group** | | | | ***Pf* group** | | | |
| --- | --- | --- | --- | --- | --- | --- | --- | --- | --- | --- | --- | --- | --- | --- | --- | --- |
| **Proteins** | **Cut-off^a^** | **J** | **AUC** | **Sens-Spec** | **Cut-off^a^** | **J** | **AUC** | **Sens-Spec** | **Cut-off^a^** | **J** | **AUC** | **Sens-Spec** | **Cut-off^a^** | **J** | **AUC** | **Sens-Spec** |
| **Ang-1** | 30.09 | 0.21 | 0.61 | 25 - 96% | 19.16 | 0.11 | 0.56 | 33 - 78% | 19.16 | 0.17 | 0.59 | 37-80% | 14.65 | 0.15 | 0.57 | 55-60% |
| **Ang-2** | 1.24 | 0.23 | 0.61 | 83 - 40% | 2.07 | 0.17 | 0.58 | 63 - 53% | 3.52 | 0.22 | 0.61 | 42-80% | 2.05 | 0.18 | 0.59 | 73-45% |
| **Tie-2** | 5.93 | 0.16 | 0.58 | 83 - 32% | 11.35 | 0.15 | 0.57 | 37 - 78% | 12.26 | 0.17 | 0.59 | 37-80% | 11.35 | 0.20 | 0.60 | 36-84% |
| **VEGF** | 0.08 | 0.14 | 0.57 | 42 - 72% | 0.16 | 0.13 | 0.57 | 52 - 61% | 0.16 | 0.16 | 0.58 | 56-60% | 0.20 | 0.14 | 0.57 | 44-70% |
| **sFlt1** | 18.71 | 0.14 | 0.57 | 50 - 64% | 28.15 | 0.17 | 0.59 | 33 - 84% | 19.58 | 0.19 | 0.59 | 53-66% | 28.11 | 0.20 | 0.60 | 27-93% |
| **VEGFR2** | 3.32 | 0.14 | 0.57 | 83 - 31% | 4.44 | 0.13 | 0.56 | 63 - 49% | 5.43 | 0.26 | 0.63 | 53-74% | 4.02 | 0.09 | 0.55 | 64-45% |
| **PlGF** | NA | 0.00 | 0.50 | 0 - 100% | 0.92 | 0.05 | 0.53 | 20 - 85% | 1.49 | 0.11 | 0.56 | 16-95% | 0.15 | 0.02 | 0.51 | 64-38% |
| **sENG** | 17.00 | 0.31 | 0.66 | 92 - 40% | 35.58 | 0.18 | 0.59 | 27 - 91% | 35.58 | 0.22 | 0.61 | 32-91% | 38.91 | 0.13 | 0.56 | 18-95% |
| **Leptin** | 30.99 | 0.13 | 0.57 | 58 - 55% | 39.99 | 0.05 | 0.53 | 27 - 78% | 39.99 | 0.09 | 0.54 | 32-77% | 28.89 | 0.09 | 0.54 | 36-72% |
| **Ang-1/Ang-2** | 3.69 | 0.11 | 0.55 | 92 - 19% | 4.40 | 0.08 | 0.54 | 72 - 36% | 4.47 | 0.07 | 0.53 | 74-33% | 4.35 | 0.14 | 0.57 | 70-44% |
| **Ang-1/Tie-2** | 3.46 | 0.14 | 0.57 | 33 - 81% | 2.14 | 0.21 | 0.60 | 47 - 74% | 2.51 | 0.18 | 0.59 | 37-81% | 2.11 | 0.29 | 0.65 | 55-75% |
| **sFlt1/PlGF** | 40.57 | 0.30 | 0.65 | 75 - 55% | 60.43 | 0.14 | 0.57 | 40 - 74% | 251.10 | 0.09 | 0.54 | 15-93% | 52.94 | 0.43 | 0.71 | 57-86% |

^a^The values of the proteins are displayed as ng/mL. The proteins were measured in maternal plasma at delivery. Non-infected (n = 165-166), Malaria (n = 156-212), *Pv* (n = 144-146), *Pf* (n = 62-66). Abbreviations: HC, head circumference; *Pv*, *P. vivax*; *Pf*, *P. falciparum*; J, Youden’s index; AUC, area under the curve; Sens, sensibility; Spec, specificity; Ang, angiopoietin; tie, tyrosine kinase; VEGF, vascular endothelial growth factor; sFlt1, soluble VEGF receptor 1; VEGFR2, soluble VEGF receptor 2; PlGF, placental growth factor; sENG, soluble endoglin.
