# Supplementary figures and images for: Association between peripheral markers in women with malaria in pregnancy and small newborns: A cross-sectional study
Source: PLOS Glob Public Health. 2025 Dec 3;5(12):e0005526. doi: 10.1371/journal.pgph.0005526 (PMC12674551; doi:10.1371/journal.pgph.0005526)

**S1 Fig.** **Flow chart of selection of pregnant women in the study.**

**
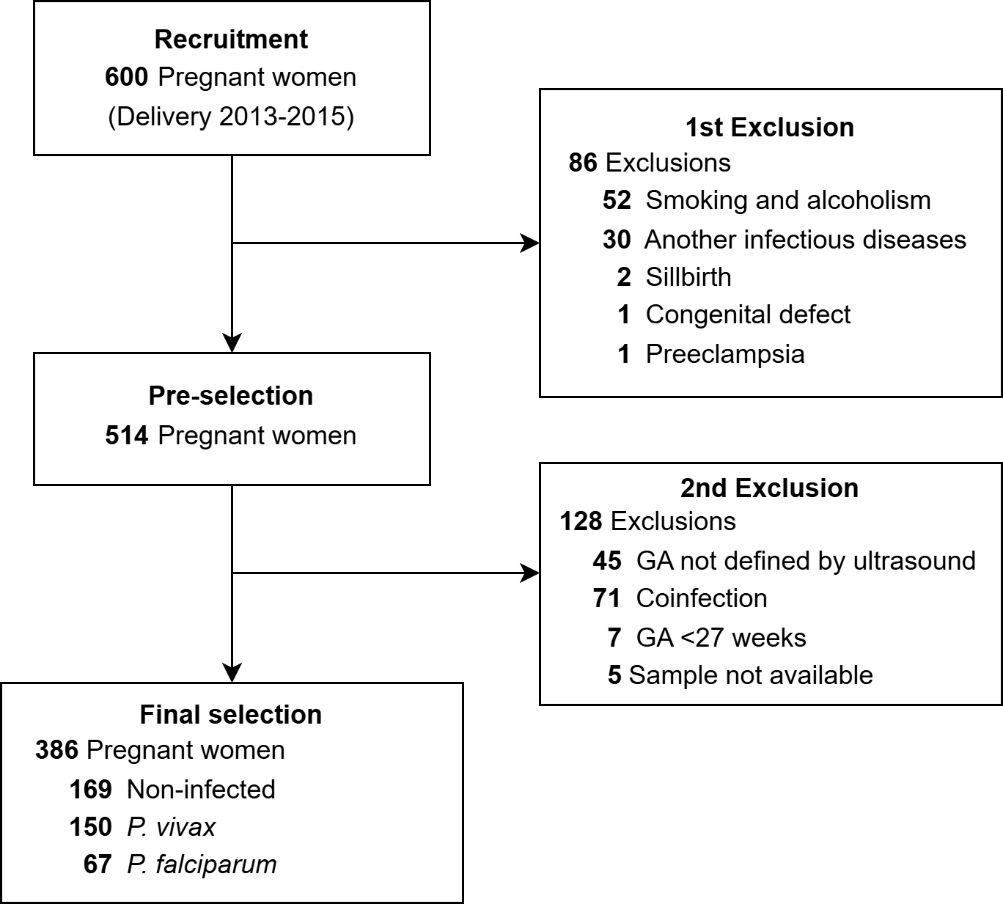
**

Abbreviations: GA, gestational age.

Supplement: S1 Fig — (DOCX) [file pgph.0005526.s008.docx]
