# Supplementary material for: Association between peripheral markers in women with malaria in pregnancy and small newborns: A cross-sectional study
Source: PLOS Glob Public Health. 2025 Dec 3;5(12):e0005526. doi: 10.1371/journal.pgph.0005526 (PMC12674551; doi:10.1371/journal.pgph.0005526)
Supplement: S2 Fig — (DOCX) [file pgph.0005526.s009.docx]

**S2 Fig. Correlation between maternal peripheral proteins with newborn growth status in the non-infected group.**


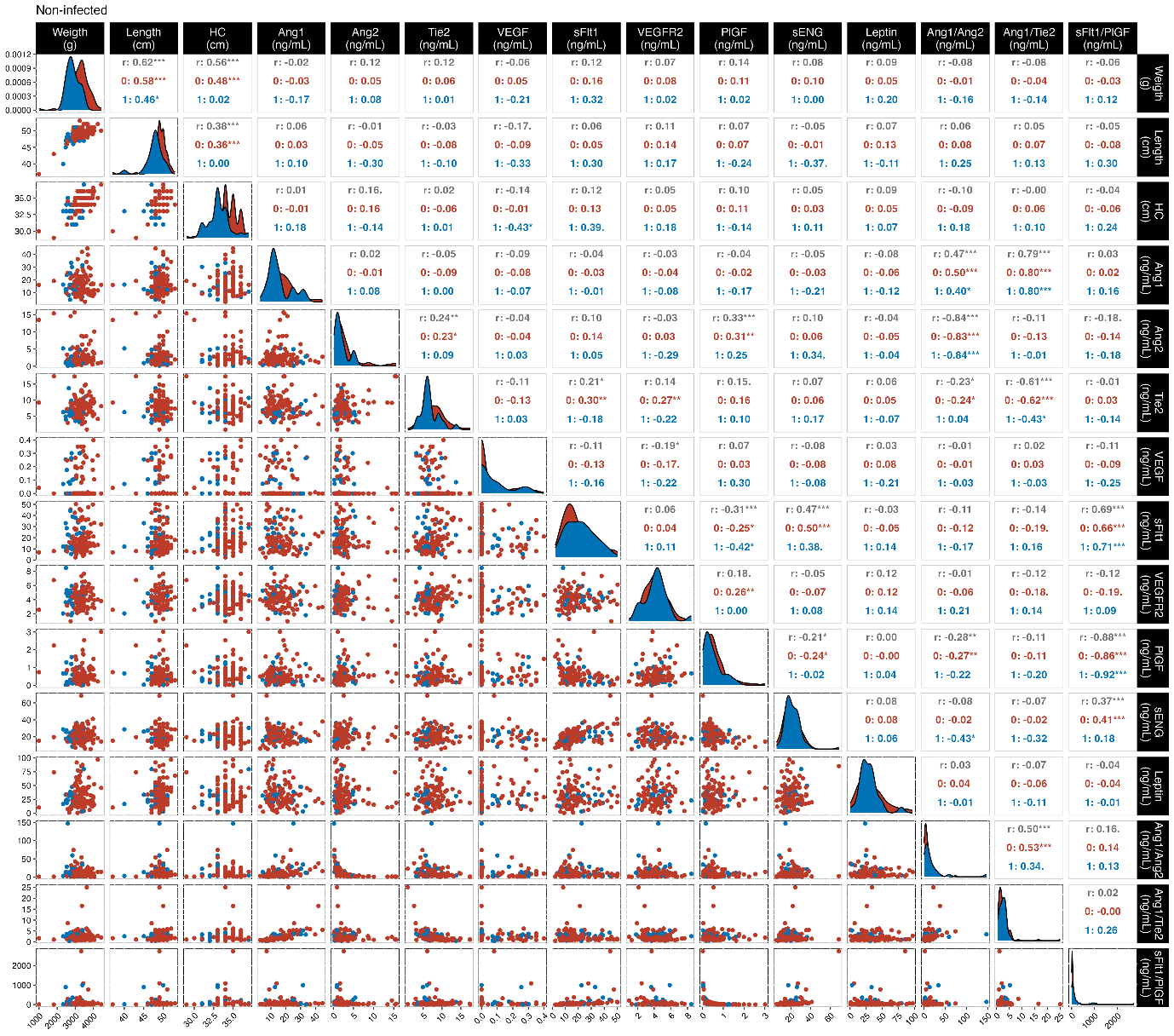


In black (r), are represented all the women from the non-infected group; in red (1), those who had newborns below the 10^th^ percentile; and in blue (0), the women who had newborns within the adequate range for their gestational age. The total number of observations for the non-infected group were n = 124, *P. vivax* n = 112, and *P. falciparum* n = 39. *p <0.05; **p <0.01; ***p <0.0001. Abbreviations: HC, head circumference; Ang, angiopoietin; Tie, tyrosine kinase; VEGF, vascular endothelial growth factor; sFlt1, receptor 1 of VEGF; R2GF, receptor 2 of VEGF; PlGF, placental growth factor; sENG, soluble endoglin.
