# Supplementary material for: Association between peripheral markers in women with malaria in pregnancy and small newborns: A cross-sectional study
Source: PLOS Glob Public Health. 2025 Dec 3;5(12):e0005526. doi: 10.1371/journal.pgph.0005526 (PMC12674551; doi:10.1371/journal.pgph.0005526)
Supplement: S3 Fig — (DOCX) [file pgph.0005526.s010.docx]

**S3 Fig. ROC curve performance of maternal peripheral proteins for newborn growth status in the non-infected group.**

**
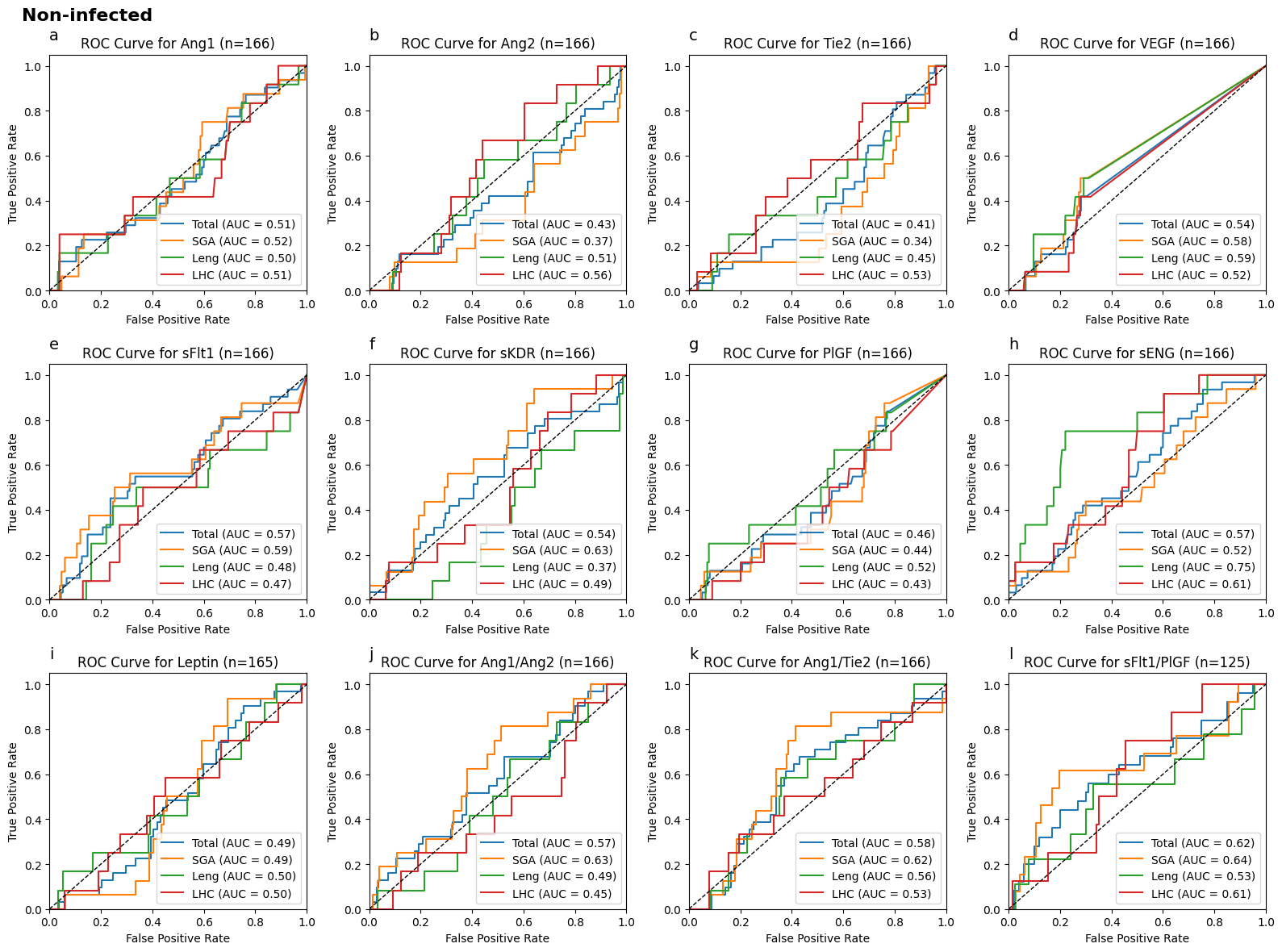
**

The units of measurement for the proteins are ng/mL. Abbreviations: SGA, small for gestational age; Leng, length; LHC, low head circumference; AUC, area under the curve; Ang, angiopoietin; Tie, tyrosine kinase; VEGF, vascular endothelial growth factor; sFlt1, receptor 1 of VEGF; R2GF, receptor 2 of VEGF; PlGF, placental growth factor; sENG, soluble endoglin.
